# Gender norms and ideologies about adolescent sexuality: A mixed-method study of adolescents in communities, south-eastern, Nigeria

**DOI:** 10.3389/fsoc.2022.810411

**Published:** 2022-09-26

**Authors:** Ifunanya Clara Agu, Chinyere Ojiugo Mbachu, Uchenna Ezenwaka, Irene Eze, Nkoli Ezumah, Obinna Onwujekwe

**Affiliations:** ^1^Health Policy Research Group, University of Nigeria, Enugu Campus, Enugu, Nigeria; ^2^Department of Community Medicine, University of Nigeria, Enugu Campus, Enugu, Nigeria; ^3^Department of Health Administration and Management, University of Nigeria, Enugu Campus, Enugu, Nigeria; ^4^Department of Community Medicine, Ebonyi State University, Abakaliki, Nigeria

**Keywords:** adolescent, gender, norms, ideology, sexuality

## Abstract

**Background:**

Sexual and reproductive health choices and behaviors of adolescents are shaped by gender norms and ideologies which are grounded in cultural beliefs. This study examined the perspectives of adolescents about the influence of gender norms and ideologies on sexuality.

**Methods:**

A cross-sectional study was undertaken in three urban and three rural communities in south-eastern Nigeria using quantitative and qualitative research methods. A modified cluster sampling procedure was used to select respondents. Data were collected from 1,057 adolescents and twelve focus group discussions with unmarried adolescents aged 13 to 18 years. For the quantitative data, univariate, bivariate and probit regression analyses were performed using Stata while the thematic framework approach was used to analyze qualitative data.

**Results:**

The dominant beliefs among adolescents are that: it is wrong for unmarried adolescents to have sex (86.4%); unmarried adolescents should abstain from sex (89.3%); consent should be obtained before sexual intercourse (89.1%); it is a girl's responsibility to ensure she does not get pregnant (66.5%), and sex should be initiated by boys (69.6%). Gender (boy or girl) was a predictor of belief in premarital abstinence (*t*-value = −3.88), belief that premarital sexual intercourse is acceptable provided contraceptive is used (*t*-value = 3.49, CI 1.14–0.49), belief that premarital sexual intercourse is wrong (*t*-value = −2.24) and, belief that sex should be initiated by boys only (*t*-value = −4.37). Adolescent boys were less likely to believe in pre-marital abstinence and less likely to believe that pre-marital sex among adolescents is wrong compared to girls. They were also more likely to believe adolescents can have sex provided contraceptive is used compared to girls. Qualitative findings revealed adolescents' beliefs that girls feel shy initiating sex and that boys experience more urge for sex hence, boys were perceived to be responsible for initiating sex. Both boys and girls experience pressure to have sex however, boys were described to experience more pressure from peers to have sex. Peer-to-peer communication, quest for material possessions and low socioeconomic conditions contribute to peer pressure to engage in sex.

**Conclusion:**

Adolescents' beliefs about sexuality underline the need to contextualize interventions to address these norms and ideologies.

## Introduction

Adolescents in sub-Sahara Africa account for 23% of the region's total population and over 30 million of them are living in Nigeria (Esiet, nd; UNICEF, 2019). Some adolescents engage in behavioral experimentation and risk-taking such as premarital sexual intercourse, unprotected sexual intercourse and other risky behaviors' that predispose them to unwanted teenage pregnancy, unsafe abortions and sexually-transmitted infections (Liang et al., 2019). The most recent demographic and health survey (DHS) in Nigeria highlighted that sexual debut occurred at 15 years for 8.6% of girls and 2.4% of boys, and 23% of sexually active adolescents in the survey engaged in unprotected sexual intercourse (National Population Commission ICF, 2019). Also, 19% of adolescent girls in the survey had already begun childbearing. Furthermore, the contraceptive prevalence rate was lowest among adolescents (15–19 years) when compared to other age categories (National Population Commission ICF, 2019). Moreover, some adolescent boys reported they had paid for sex in the 6 months preceding the survey and none of these boys used a condom in the last sexual encounter (NDHS, 2019).

A vast proportion of adolescents reach sexual maturity before attaining the social skills, and the mental and emotional maturity required to comprehend the consequences of sexual activity (Isiugo-Abanihe et al., 2015). Hence, their vulnerability to sexual and reproductive health (SRH) risks. Many preventable and life-threatening SRH problems are acquired during adolescence as they begin to explore their sexuality (Isiugo-Abanihe et al., 2015). In some cultures, sexuality remains a myth or enigma for adolescents, and limited discussions about sexuality or sex-related matters affect their perceptions of sexuality (Kar et al., 2015; Mbachu et al., 2020a). Although sexuality is a natural component of human nature that influences one's thoughts, sexual orientation, feelings and physical health, adolescents need to acquire managerial skills for this powerful developmental force. Human sexuality encompasses an individual's sexual interest including sexual fantasies, attitudes and values related to sex and sexual orientation (Lumen learning courses, nd; Center for Young Women's Health, 2018). Sexual orientation involves having erotic or sexual feelings for people of the same gender, a different gender, or more than one gender. However, the focus of this study is sexual orientation toward a different gender that involves a male and a female.

Although dating relationships are mostly perceived as the primary avenue for sexual exploration among adolescents, sexuality could be expressed in several ways in different relationship contexts. Sexuality could be expressed in non-dating relationships, individually, in casual encounters, through hugging, holding hands and among others (Ott, 2010). Sexual behavioral practices are believed to be determined by biological components however, some authors affirm that sexual behavior is largely molded by the environment (Kar et al., 2015; Lumen learning courses, nd; Challa et al., 2018). Beliefs and norms about sexuality prevail in society, and among adolescents, and these could influence adolescents' SRH choices. Beliefs broadly refer to a person's values, principles, assumptions, and expectations (Cole, 2019). In society, social norms are informal rules that govern behavior in groups and societies (Bicchieri et al., 2018). Norms are customary or accepted standards and ideologies, which provide a set of values and beliefs that guide attitudes and behaviors. Ideologies and norms play a powerful role in shaping thoughts, actions, interactions, and they are also instrumental in organizing the functioning of a given society (Bicchieri et al., 2018; Cole, 2019; Barrett et al., 2021).

At an early age, young people adopt and act on expected societal roles and norms about sexuality and relationships (Vu et al., 2017). Traditional gender roles pose restricted sexuality in most females as they are commonly perceived as subordinate to males, nurtured to be obedient and take a passive submissive role in any sexual relationship whereas, males are fostered to be masculine in gender, demonstrating autonomous, brave assertive and dominating characteristics (Lawoyin and Kanthula, 2010; Macia et al., 2011; Muralidharan et al., 2015; Ninsiima et al., 2018; Lewis et al., 2021; Zimmerman et al., 2021). Some studies have shown that subscribing to these conventional gender norms, and beliefs, adolescents experience difficulty engaging in successful and satisfying relationships which could further predispose them to indulge in some risky sexual behaviors (Capurchande et al., 2016; Casique, 2019; Lewis et al., 2021). This indicates that gender norms and ideologies play a significant role in understanding adolescent sexual attitudes and behaviors.

Adolescents' approach toward ideologies and gender norms about sexuality could either favor or limit them from expressing their sexual desires. Research has shown that because of the strong ideologies and norms that encourage women/girls to be submissive and remain virgins until they are married, it may be difficult for them to negotiate safe sex or access treatment services when necessary as they may be subjected to stigma (Little and McGivern, 2014). In some cultures, males are believed to be more sexually active than females and research confirm that women think about sexual intercourse on an average of 10 times per day compared to 19 times per day for men (Fisher et al., 2011). As previously described, some studies have focused on understanding social and environmental influences of adolescents' sexuality (Kar et al., 2015; Challa et al., 2018), while some others reported differences between men's and women's sexuality (Fisher et al., 2011; Little and McGivern, 2014).

Studies examining socio-cultural notions of sexuality and sexual relationships in Nigeria have shown that these notions are shaped by deep-seated patriarchal norms and religious beliefs in male superiority (Odimegwu et al., 2010; Abayomi and Olabode, 2013; Fakunmoju et al., 2016). Moreover, the entrenchment of male dominance in sexual (and other social) relationships is mediated by patriarchal ideologies, including perceptions of male privilege, that are embedded in religious beliefs (Amusan et al., 2017; Ajayi et al., 2022). Although it is argued that these notions may facilitate unsafe sexual practices and sustain the cultural devaluation of women among adolescents (Izugbara, 2005), there is paucity of empirical studies on this topic among adolescents in Nigeria.

Considering adolescents' vulnerability to sexuality-related SRH issues, an understanding of their notions of gender norms and beliefs about their sexuality is the paramount and first step for strategic intervention(s). Moreover, gender norms and beliefs may vary in urban and rural areas seeing that geographic locations may be reflective of cultural differences (Rakauskas et al., 2009). However, there is scarcity of evidence to support or refute geographic differences in sexual norms among adolescents and young people.

We undertook a study in Ebonyi State, Nigeria, to identify the prevailing ideologies and gender norms about adolescent sexuality. This was part of a wider study that examined the situation and determinants of adolescents' SRH in urban and rural areas in the State. This paper, however, focuses on the perspectives of adolescents alone. This knowledge could contribute to underlining the need for comprehensive sexuality education interventions that are contextualized to address gender norms and ideologies among adolescents.

## Materials and methods

### Study setting and design

This study was carried out in three urban and three rural communities in Ebonyi State, south-east Nigeria. The state has over 6 million inhabitants with a fertility rate of 5.4% (NDHS, 2019). More than 40% of Ebonyi state's total population are under the age of 15 years (USAID Health Policy Plus., 2017).

The study sites were spread across six local government areas (LGAs) that were selected from the three senatorial zones in the state. In each senatorial zone, two (2) LGAs were purposively selected for the study to reflect the state governments' prioritization of adolescent health interventions. The six study communities were also selected purposively. These selected LGAs and communities were prioritized by the State government for interventions and the key stakeholders also listed them as having the highest abortion and unwanted teenage pregnancy rates in the State.

This was an exploratory cross-sectional study of adolescents. We employed a mixed-method convergent parallel design in which quantitative and qualitative research methods were used simultaneously to obtain complementary information. The quantitative method involved head of household and adolescent interviewer-administered surveys, while the qualitative method employed focus group discussions.

### Study participants and sampling

The study was carried out among in-school and out-of-school unmarried adolescent boys and girls aged 13 to 18 years who were living in the selected urban and rural study areas. The quantitative study population were unmarried adolescents aged 13 to 18 years and residing in selected households in the study communities. To achieve a 5% precision at 95% confidence interval for a population >100,000 a minimum sample size of 400 was determined (Glenn, 1992). This was doubled to enable subgroup analysis of data and increased to over 1,000 for robustness and to account for incomplete responses and errors. Participants were selected through a modified cluster sampling technique. A cluster was defined as an autonomous community being governed by a traditional ruler. Households were consecutively selected using the nearest public facility identified from the main entrance as the starting point. In selected households, all eligible adolescents were invited to participate in the study.

For the qualitative study, out-of-school adolescents in apprentice workshops were purposively selected from a subset of respondents (older adolescents aged 15–18 years) who appeared to be informed during the study survey. The in-school adolescents were randomly selected from public secondary schools in each study community. Then, they were all invited to participate in focus group discussions (FGDs). A detailed description of eligibility criteria and participant selection procedure can also be found in a previously published manuscripts (Mbachu et al., 2020a,b).

### Ethical consideration

Ethical clearance was obtained before community entry from the Health Research Ethics Committee of University of Nigeria Teaching Hospital with reference number NHREC/05/01/2008B-FWA00002458-IRB00002323 and also approval from the Research and Ethics Committee of Ebonyi State Ministry of Health. Informed written consents were obtained from parents/guardians of adolescents aged 13 to 18 years whereas those aged 18 years gave consent for themselves. In addition to parental consent, informed consent was sought from all (including adolescents aged 13 to 18 years) eligible participants having informed them of the purpose of the research project, their rights as participants, potential risks and benefits of participation, and measures to ensure confidentiality of information. Documentation of informed consent or assent was through signature or thumbprint, in the case of low literacy, of all eligible participants and/or their parents/guardians, where applicable.

### Data collection

The quantitative data was collected from 1,057 adolescents from selected households. The adolescent questionnaire was adapted from the WHO illustrative questionnaire for interview surveys with young people (Cleland, 2001). The household questionnaire was specifically designed for this study to collect information on household expenditure patterns from the head of households. The questionnaires were pre-tested in a contingent state among heads of households and in-school and out-of-school adolescent boys and girls, respectively. Data were collected for 10 days by fifty-four research assistants who were recruited and trained for 5 days to be able to administer paper and electronic copies (SurveyCTO) of the questionnaire and to be familiar with the study questions. Each pair of research assistant administered both paper and electronic copies of the questionnaire concurrently through a face-to-face interviewer-administered approach. Information on the completed paper-questionnaire were individually matched with the corresponding electronic-questionnaire. These information were double-checked before and after uploading data to the server.

To collect qualitative data, one focus group discussion for boys and another for girls were conducted in each community. Hence twelve focus group discussions comprising six groups for boys and girls, respectively were conducted in the six selected communities using a pre-tested interview guide. The discussions were conducted according to participants' language preferences either in English or Igbo language. Permission to audio record each discussion was sought before the discussion. In each FGD, a moderator and a note-taker facilitated the discussion comprising of 8 to 10 participants. Written consent was obtained from each participant before discussion and parental consent was also obtained for participants <18 years. The purpose of the study was clearly stated, roles and rights of participants were provided and confidentiality of their information assured before obtaining consent. Each FGD discussion lasted for about 60–70 min on average.

### Data analysis

A total of 1,045 questionnaires that were completely and correctly filled were analyzed (response rate of 95%). Data were analyzed using Stata software. Descriptive analysis was performed and weighted proportions were reported for categorical variables. The test for association of variables was performed, Chi-square (χ^2^) and *p*-values were reported for the multi-way tables. The frequency and proportion were carried out to show unconditional differences in gender norms and beliefs about adolescent sexuality, socioeconomic and demographic characteristics such as age, gender, schooling, place of residence (urban or rural) and wealth index.

Binominal logistic regression analysis was performed to identify socioeconomic and demographic predictors of gender norms and ideologies about sexuality. The analysis was extended by isolating specific determinants of gender norms and gender ideologies about sexuality, while taking into consideration, variations across individual socio-economic and demographic characteristics under a regression framework. The primary aim is to understand if there is an interaction between the independent variables and the different outcome variables of interest. Our multivariate regression model can be specified parsimoniously as:


(1) $$\begin{array}{l} Y_{i}=\beta_{0}+\beta_{1}X_{i}+\mu_{i} \end{array}$$

Where *Y*_*i*_, the outcome variable for individual *i*, which is a dummy variable that uses the value of 1 if an individual respondent agrees to a given gender norm or gender ideology and 0 disagree. *X*_*i*_ is a vector of control variables for individual *i*, this includes gender, schooling, place of residence (urban or rural), work status and wealth index. The error term, μ_*i*_, is taken to be normally distributed.

The outcomes of interest for gender ideologies include; belief that it is wrong for unmarried adolescents to have sex; belief in premarital abstinence; belief that adolescents can have sex provided they use contraceptives; and belief that consent should be obtained from the sexual partner before sexual intercourse. The outcomes of interest for gender norms were; belief that a boy has to force a girl to have sex; belief that sex should be initiated by boys only or by either gender (boy and girl); and belief that it is the girl's responsibility to ensure she does not get pregnant following sexual intercourse. Statistical significance was set at *p* ≤ 0.05 and a confidence level of 95%.

The household wealth index was calculated using per capita household consumption patterns based on food and non-food expenditure for 1 year. The per capita household consumption was used to classify households into socio-economic quintiles, Q1 to Q5, where Q1 refers to poorest households and Q5 richest households. All the quantitative materials including the datasets can be found in the repository, UK Data Service 10.5255/UKDA-SN-854374 (Mbachu et al., 2020b,c).

**Figure 1:**
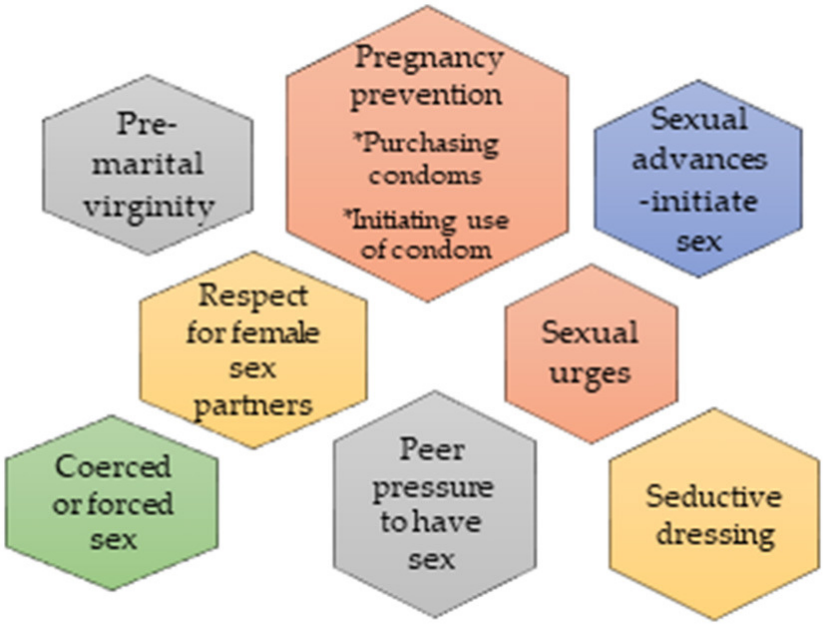
Coding scheme for gender norms and ideologies about adolescent sexuality.

The audio files were transcribed verbatim and translated into English where necessary. All transcripts were anonymously coded and data were kept in a password, protected laptop. A thematic framework approach was used to analyze the data. Two independent researchers were given the richest transcript to read several times and gain full insight into the text pattern. Texts were double coded and checked for differences and similarities while discrepancies were resolved. In initial coding, emerging themes and discrepancies were reviewed by the research team leading to a final coding framework. Then, the final coding framework was used to manually code all the transcripts including the one that was used to generate themes. The thematic areas in the final coding framework are shown in Figure 1.

### Quality of data and findings

In order to minimize researchers' bias and ensure more disciplined subjectivity in qualitative data collection and analysis, the following were done, (i) FGDs were moderated by experienced qualitative researchers who received additional training on how to maintain a neutral stance during the discussions; (ii) researchers were trained to document (and report) any personal biases and nuances in their field notes after each FGD; (iii) the initial themes were generated deductively by two independent researchers; (iv) each transcript was coded by two independent researchers; (v) discrepancies in the generation of themes and coding of transcripts were resolved through consensus.

## Results

### Demographic and socio-economic characteristics of the participants

Among 1,045 survey participants, 50.7% were from urban areas while 49.3% were from rural areas. There were 57.2% girls and 42.8% boys in the survey. Their mean age was 15.4 years (CI, 15.3–15.5). Fourteen-year-olds constituted the highest number of adolescents in the survey (20.9%), while seventeen-year-olds were the least in number (12.9%). The majority (92.4%), of the adolescents, were currently in school at the time of the survey. Slightly half (51%) of the adolescents reported that they do work for payment. The wealth index distribution of adolescents into quintiles is also shown in Table 1. Q1 represents the number and percent of adolescents in the poorest household quintile while Q5 represents those in the richest household quintile.

**Table 1:** Demographic characteristics of surveyed adolescents.

**Variables (*N* = 1,045)**	**Frequency (*n*)**	**Weighted percent (%)**
**Place of residence**	
Urban	551	50.7
Rural	494	49.3
**Gender**	
Female	598	57.2
Male	447	42.8
**Age in single years**	
13	180	17.4
14	219	20.9
15	162	15.5
16	151	14.5
17	136	12.9
18	197	18.8
**Schooling status**	
In-school	966	92.4
Out-of-school	79	7.6
**Work status (working for pay)**	
Working for pay	543	51.96
Not working for pay	502	48.0
**Wealth index**	
Q1	224	21.9
Q2	211	20.6
Q3	214	20.0
Q4	198	18.8
Q5	197	18.6

### Adolescents' ideologies about their sexuality

The findings showed that 86.4% of adolescents in the survey believed it is wrong for unmarried adolescents to have sex; 89.3% believed that unmarried boys and girls should abstain from sex; 18.4% believed it is alright for adolescents to have sex provided they use contraceptives, and 89.1% believed that consent should be gotten from a partner before sexual intercourse. The analysis of gender norms about adolescent sexuality shows that 10% of adolescents in the survey believed that a boy has to force a girl to have sex; 66.5% of adolescents believed that it is a girl's responsibility to ensure that she does not get pregnant after sexual intercourse. Their opinions about who should initiate sexual intercourse show that the majority of them, 69.6%, think that sex should be initiated by boys, and 19.5% think that sex could be initiated by either the boy or the girl (Table 2).

**Table 2:** Ideologies and gender norms about adolescent sexuality.

**Variables (*N* = 1,045)**	**Frequency (*n*)**	**Percent (%)**
**Ideologies about adolescent sexuality and sexual behaviors**	
Believes it is wrong for unmarried adolescents to have sex	902	86.4
Believes boys and girls should abstain from sex until they marry	934	89.3
Believes consent should be obtained from the sexual partner before intercourse	195	18.4
Believes it is alright for adolescents to have sex provided they use contraceptives	931	89.1
**Gender norms about adolescent sexuality and sexual behaviors**	
Believes a boy has to force a girl to have sex with him	108	10
Believes sex should be initiated by boys only	728	69.6
Believes sex could be initiated by either the boy or the girl	202	19.5
Believes it is the girls' responsibility to ensure she does not get pregnant	696	66.5

Place of residence and sex/gender had statistically significant correlations with belief in abstinence, (*p* < 0.001). Adolescent girls than boys, and those who reside in urban areas as compared to rural dwellers are more likely to believe that boys and girls should abstain from sex until they marry. Those who work for payment as compared to those who do not work to receive income are more likely to believe that boys and girls should abstain from sex until they marry. Sex/gender, schooling and work status also had significant correlations with respondents' belief that sex is permitted among adolescents as long as contraceptives are used, (*p* ≤ 0.01). Boys and out of school adolescents are more likely to believe that sex is permitted among adolescents as long as contraceptives are used as compared to girls and in-school adolescents, respectively. Wealth index correlated significantly with belief that unmarried adolescents engaging in premarital sex are wrong (*p* = 0.02) (Table 3).

**Table 3:** Socio-demographic disaggregation of ideologies about adolescent sexuality.

	***N***	**Believes it is wrong for unmarried adolescents to have sex *n* (%)**	**Believes boys and girls should abstain from sex until they marry *n* (%)**	**Believes it is alright for adolescents to have sex provided they use contraceptives *n* (%)**	**Believes consent should be obtained from the sexual partner before intercourse *n* (%)**
**Place of residence**	
Rural	494	415 (84)	423 (85.6)	98 (19.8)	434 (87.8)
Urban	551	487 (88.6)	511 (92.8)	97 (17.1)	497 (90.2)
*χ^2^* (*p*-value)		2.43 (0.09)	8.15 (<0.001)*	2.30 (0.10)	0.86 (0.42)
**Sex/Gender**	
Female	598	529 (88.4)	555 (92.7)	91 (15)	545 (91)
Male	447	373 (83.6)	379 (84.5)	104 (22.9)	386 (86.4)
*χ^2^* (*p*-value)		2.58 (0.08)	8.88 (<0.001)*	5.31 (0.01)*	5.31 (0.01)*
**Schooling**	
In-school	79	835 (86.5)	865 (89.4)	168 (17.1)	859 (89)
Out-of-school	966	67 (85)	69 (87.2)	27 (33.9)	72 (90.9)
*χ^2^* (*p*-value)		0.33(0.72)	0.50 (0.60)	6.85 (0.01)*	1.43 (0.24)
**Work status**	
Working for pay	543	416 (89.50)	427 (85.06)	109 (21.71)	449 (89.44)
Not working for pay	502	486 (89.50)	507 (93.37)	86 (15.84)	482 (88.77)
*χ^2^* (*p*-value)		10.54 (0.005)*	20.57 (0.000)*	6.24 (0.04)*	0.20 (0.91)
**Wealth index**	
Q1 (poorest)	224	186 (83)	194 (86.6)	41 (18.3)	195 (87)
Q2	211	183 (86.7)	191 (90.5)	35 (16.6)	189 (89.6)
Q3	214	179 (83.4)	190 (88.8)	51 (23.8)	186 (86.9)
Q4	198	167 (84.3)	178 (89.9)	34 (17.2)	177 (89.4)
Q5 (richest)	197	186 (94.4)	180 (91.4)	34 (17.3)	183 (92.9)
*χ^2^* (*p*-value)		18.17 (0.02)*	8.68 (0.37)	7.45 (0.49)	6.34 (0.61)
Total	1,045	902 (86.4)	934 (89.3)	195 (18.4)	931 (89.1)

*p-value < 0.05. The bold values indicates the statistical significant which is set at *p-value ≤ 0.05.

### Gender norms about adolescent sexuality

Statistically, a significant association was observed between the place of residence and the belief that girls should be forced to have sex, (*p* < 0.001), and that girls are responsible for ensuring the use of contraceptives during sexual intercourse (*p* = 0.01). Sex/gender was found to have a significant correlation with respondents' beliefs that sex should be initiated by boys only (*p* < 0.001). Work status has a significant correlation with respondents' beliefs that sex should be initiated the boy or the girl (*p* < 0.001). Wealth index correlated with belief that sex could be initiated by either the boy or the girl, (*p* = 0.02) (Table 4).

**Table 4:** Socio-demographic disaggregation of gender norms about adolescent sexuality.

	**N**	**Believes a boy has to force a girl to have sex with him *n* (%)**	**Believes sex should be initiated by boys only *n* (%)**	**Believes sex could be initiated by either the boy or the girl *n* (%)**	**Believes it is the girls' responsibility to ensure she does not get pregnant *n* (%)**
**Place of residence**	
Rural	494	32 (6.5)	334 (67.6)	108 (21.9)	309 (62.5)
Urban	551	76 (13.5)	394 (71.6)	94 (17.2)	387 (70.3)
*χ^2^* (*p*-value)		7.26 (<0.001)*	1.98 (0.16)	3.59 (0.06)	4.57 (0.01)*
**Sex/Gender**	
Female	598	63 (10.2)	445 (74.4)	105 (17.7)	415 (69.3)
Male	447	45 (9.9)	283 (63.3)	97 (21.9)	281 (62.7)
*χ^2^* (*p*-value)		0.06 (0.94)	15.01 (<0.001)*	2.79(0.09)	2.83(0.06)
**Schooling**	
In-school	79	100 (10.1)	671 (69.4)	187 (19.5)	643 (66.4)
Out-of-school	966	8 (9.8)	57 (72)	15 (19.2)	53 (67)
*χ^2^* (*p*-value)		3.04 (0.05)	0.23 (0.63)	0.01 (0.95)	0.46 (0.63)
**Work status**	
Working for pay	543	52 (10/36)	352 (92.39)	107 (88.43)	331 (65.94)
Not working for pay	502	56 (10.31)	376 (92.84)	95 (68.84)	365 (67.22)
*χ^2^* (*p*-value)		1.85 (0.40)	0.06 (0.61)	14.41 (<0.001)*	3.57 (0.17)
**Wealth index**	
Q1 (poorest)	224	21 (9.4)	154 (68.7)	54 (24.1)	137 (61.2)
Q2	211	18 (8.5)	151 (71.6)	27 (12.8)	149 (70.6)
Q3	214	34 (15.9)	153 (71.5)	36 (16.8)	136 (63.5)
Q4	198	18 (9.1)	143 (72.2)	39 (19.7)	135 (68.2)
Q5 (richest)	197	17 (8.6)	126 (63.9)	46 (23.3)	139 (70.6)
*χ^2^* (*p*-value)		11.90 (0.15)	4.43 (0.35)	11.97 (0.02)*	11.6 (0.17)
Total	1,045	108 (10)	728 (69.6)	202 (19.5)	696 (66.5)

*p-value < 0.05. The bold values indicates the statistical significant which is set at *p-value ≤ 0.05.

The multivariate analysis of adolescents' ideologies about sexuality and sexual behaviors is presented in Table 5. The dependent variables were the belief that it is wrong for unmarried adolescents to have sex; belief in premarital abstinence; belief that adolescents can have sex provided they use contraceptives; and belief that consent should be obtained from the sexual partner before sexual intercourse. Predictors of respondents' belief that it is wrong for unmarried adolescents to have sex include sex/gender (*t*-value = 1.97, CI 0.00–0.39) and work status (*t*-value = −2.24). Sex/Gender was also found to predict respondents' belief in abstinence from pre-marital sex (*t*-value 3.89, CI−0.63 to −0.21), and their belief that adolescents can have sex provided contraceptive is used (*t*-value = 3.52). Boys were less likely to believe in pre-marital abstinence and to believe that pre-marital sex among adolescents is wrong when compared to girls. They were also more likely to believe that adolescents can have sex provided contraceptive is used compared to girls. Place of residence and working status were also found to predict respondents' believe in premarital abstinence. In-school adolescents (*t*-value = −3.52) compared to out of school adolescents are less likely to believe that adolescents can have sex provided contraceptive is used (Table 5).

**Table 5:** Probit regression of factors associated with ideologies about adolescent sexuality and sexual behaviors.

**Demographic variables**	**It is wrong for unmarried adolescents to have sex**			**Boys and girls should abstain from sex until they get married**			**Adolescents can have sex provided they use contraceptives**			**Consent should be obtained from the sexual partner before intercourse**		
	***t*-value**	**SE**	**95% CI**	***t*-value**	**SE**	**95% CI**	***t*-value**	**SE**	**95% CI**	***t*-value**	**SE**	**95% CI**
Place of residence (urban)	1.97*	0.10	0.00–0.39	3.20*	0.11	0.14–0.56	−0.91	0.09	−0.25–0.09	1.54	0.12	−0.05–0.40
Sex/Gender (male)	-2.24*	0.10	−0.42 to −0.03	-3.88*	0.12	−0.63 to −0.21	3.49*	0.09	1.14–0.49	−0.74	0.12	−0.31–0.14
Schooling status(in-school)	0.43	0.18	−0.28–0.43	0.55	0.19	−0.27–0.49	-3.52*	0.15	−0.85 to −0.24	−1.52	0.27	−0.94–0.11
Work status (working for pay)	-2.24*	0.11	−0.44 to −0.03	-2.95*	0.12	−0.58 to −0.12	1.25	0.10	−0.07–0.31	0.99	0.12	−0.12–0.37

Statistical significance: *p < 0.05; variables in the bracket are the groups of interest (e.g., female = 1). The bold values indicates the statistical significant which is set at *t-value ≥ 1.96.

Regarding predictors of gender norms about adolescent sexuality and sexual behaviors, the dependent variables were belief that a boy has to force a girl to have sex; belief that sex should be initiated by boys only or by either gender (boy and girl); and belief that it is the girl's responsibility to ensure she does not get pregnant following sexual intercourse. Compared to adolescents in rural areas, those in urban areas were more likely to believe that a boy has to force a girl to have sex (*t* = 3.90, CI, 0.21–0.65). Respondents' believes that sex should be initiated by the boy alone was predicted by their sex/gender (*t* = −4.37). Adolescent boys in the survey were less likely to believe that sex should be initiated by boys alone, compared to girls. Working for pay (*t* = 3.65) increases the likelihood of believes that sex could be initiated by either the boy or the girl (Table 6).

**Table 6:** Probit regression of factors associated with gender norms about adolescent sexuality and sexual behaviors.

**Demographic variables**	**Believes a boy has to force a girl to have sex with him**			**Believes sex should be initiated by boys only**			**Believes sex could be initiated by either the boy or the girl**			**Believes it is the girls' responsibility to ensure she doesn't get pregnant**		
	***t*-value**	**SE**	**95% CI**	***t*-value**	**SE**	**95% CI**	***t*-value**	**SE**	**95% CI**	***t*-value**	**SE**	**95% CI**
Place of residence (urban)	3.90*	0.11	0.21–0.65	0.38	0.14	−0.22–0.33	−1.75	0.17	−0.65–0.04	1.89	0.17	−0.01–0.32
Sex/Gender (male)	−0.23	0.11	−0.23–0.19	-4.37*	0.14	−0.88 to −0.34	0.22	0.18	−0.31–0.38	−1.70	0.82	−0.30–0.02
Schooling status (in-school)	−0.78	1.97	−0.54–0.23	0.72	0.24	−0.30–0.66	−1.47	0.50	−1.72–0.24	−0.56	0.16	−0.40–0.22
Work status (working for pay)	0.52	0.11	−0.16–0.28	1.43	0.15	−0.08–0.50	3.65*	0.20	0.34–1.13	−0.24	0.09	−0.19–0.15

Statistical significance: * *p* < 0.05; Variables in the bracket are the groups of interest (e.g., female = 1). The bold values indicates the statistical significant which is set at *t-value ≥ 1.96.

### Qualitative findings

#### Discussion topics

(A)Pre-marital virginity
(B)Pregnancy prevention responsibility-during or after sexual intercourse
  (i) Purchasing condoms
  (ii) Initiating use of a condom
(C)Sexual advances - initiating sex
(D)Respect for female sex partners
(E)Sexual urges
(F)Coerced or forced sex
(G)Peer pressure to have sex
(H)Seductive dressing.

#### Premarital virginity (is premarital virginity more important or more required for girls than boys?)

There are variations in adolescents' responses about boys and girls maintaining premarital virginity. Although both boys and girls feel that premarital virginity is expected of boys and girls, some feel it is more important for girls to remain virgins before marriage. Their reasons for supporting premarital virginity for girls are as follow:

(i) The cultural norm and Christian values demand that girls maintain their virginity before the marriage.
(ii) To enable girls to have love and confidence of potential spouses, which is expected to make the husbands respect them, and be proud of them.
(iii) Because girls bear the brunt of the consequences of losing virginity before marriage, which could lead to, unwanted pregnancy, dropping out of school if pregnant, and distraction in academic work.
(iv) To avoid contracting sexually transmitted infections.
(v) Loss of virginity affects a girl's marriage options because nobody wants to marry a girl who is not a virgin.
(vi) Unwanted pregnancy has adverse consequences for girls.

These expressions are substantiated by the following quotes;

“*Is for girls because when you are a virgin and you get married, your husband will love you better than another person. When he goes out with his friends, he will be telling them do you know that my wife is the best…” (R1, ADABF, female adolescent)*.

“*A girl is supposed to be a virgin because if a man comes to marry her and finds out that she has a baby the man will leave her and check elsewhere.” (R11, ADIKM, male adolescent)*

“*What I think about boys and girls having sex before marriage is that if you start having sex with a boy and you are not married to him the boy may get you pregnant and dump you”. (R4, ADEZF, female adolescent)*

Concerning support for pre-marital virginity for both boys and girls, some male respondents feel that boys and girls are expected to be virgins to avoid adverse consequences such as contracting STIs. Quotes buttressing this expression follow.

“*It (premarital virginity) is required for both boys and girls because if one has sex before marriage, he or she may contract HIV …” (R5, ADEZM male adolescent)*

“*It is good for both of them to be virgins because after marriage they may love each other. But when they break their virginity, the lady may hate the man and the man may hate the lady also” (R11, ADEZM, male adolescent)*

#### Responsibility to prevent pregnancy during or after sexual intercourse

##### Purchasing condoms

Many respondents feel that both boys and girls should buy condoms to ensure it is available when they want to have sexual intercourse to avoid unwanted pregnancy and sexually transmitted infections. “Both of them, because both of them are afraid of pregnancy.” (R5, ADEZM)

Concerning boys, the perception is that they should purchase condoms to ensure that the girls they have sex with do not become pregnant. Supporting quotes include the following;

“*As for me, I can say that it is the boy because if you want to have sex you will buy the condom to avoid unwanted pregnancy” (R6, ADIKM male adolescent)*.

“*The Boy... Some would say let them use it so that the girl will not get pregnant. Some will say I don't have money to abort the baby” (R5 ADEZF, female adolescent)*.

While in the case of girls, the responses are that girls should buy condoms to protect themselves from contracting sexually transmitted infections because they can easily contract diseases. Females are expected to buy condoms because they want to prevent unwanted pregnancies. Furthermore, participants conveyed the notion that some males dislike using condoms during sexual intercourse therefore, the female partner should be the person to purchase condom to ensure that condom is available during sex. These expressions are supported by the following quotes;

“*Girls are the ones that will buy condoms because they are the ones the sperm is going into their body” (R3, ADIKM, male adolescent)*.

“*Females because some males don't like using a condom, the reason being that when they are using a condom, they don't enjoy the sex” (R5, ADABF, female adolescent)*.

However, most boys share the view that both boys and girls should purchase condoms because they do not want to contract STIs, especially when they engage in casual sexual intercourse; that a girl needs to have her condom when she wants to have sex even if the boy does not have any at hand to avoid pregnancy. These reasons are expressed in the following quotes;

“*Both of them should have the condom because they have not known each other so that they will not contract disease” (R3, ADIKM, male adolescent)*.

“*Girls or boys should buy a condom so that when the boy is not with his own the girl will be with her own and when they have sex the girl will not be pregnant” (R5, ADIKM, male adolescent)*.

“*Using a condom is for a boyfriend and girlfriend is because you don't know how the girl is doing before” (R6, ADIKZ, male adolescent)*.

According to some female respondents, a major constraint a girl may have in purchasing a condom is that she will be afraid the boy will perceive that she is scared of contracting an infection from him and also because some males do not like to use a condom. Illustrating quotes include the following;

“*The girls cannot buy condoms because if the girl buys condom the boy will be thinking that the girl does not want to contract disease” (R4, ADEZF, female adolescent)*.

“*Females do not buy it because some males don't like using condoms reason being that when they are using a condom, they don't enjoy sex” (R6, ADAFF, female adolescent)*.

##### Initiating use of a condom

Many of the male and female respondents think it is the girl's responsibility to protect herself from unwanted pregnancy because she experiences the shame of unwanted pregnancy.

Illustrative quotes follow;

“*It is the girl's responsibility because the disgrace will be felt more by her family*” (R1, ADABF female adolescent).

“*…the girl because she does not want to get pregnant and when the boy denies her and there is nothing she can do, she will be the one to suffer” (R3, ADIKF, female adolescent)*.

“*I can say that it is the girls because the consequences would be borne by the girls” (R6, ADIKM, Male adolescent)*.

“*It is the girl. As I am now, I may see a girl not that I love her but, I love her because of what she has. I may even go near to her and have sex with her and then I will leave her, and if she becomes pregnant that one does not concern me” (R12, ADIKM, male adolescent)*.

Regarding the responsibility of the boy, a few male and female respondents also feel it is the boy's responsibility to initiate the use of condom during sexual intercourse to prevent them from contracting STIs, and ensure the girl does not get pregnant because he will be compelled to take responsibility of the girl if pregnancy occurs. The following quotes substantiate the responses.

“*It is the responsibility of the boy to try all possible means to prevent pregnancy. If she, unfortunately, gets pregnant the parents will ask her the very person that impregnated her; all the blame has to go back to the very person that impregnated her. Now when they get to the boy's family the boy will be regretting why he failed to protect himself” (R1, ADEZM, male adolescent)*.

“*It is the boy because the boy will make use of the condom to prevent certain diseases (R3, ADIKM, male adolescent)*.

“*…the boy because when he impregnates the girl…he has nothing to offer when the parents of the girl tell him to take her as his wife” (R4, ADIKF, female adolescent)*.

#### Sexual advances-initiating sexual intercourse

The majority of the male and female respondents think that boys initiate sexual advances because girls are shy to do that and because males have a greater urge for sex. Only a few female respondents said that girls also initiate sex.

“*It is the boy because the girl will be feeling shy to talk about it” (R2, ADABF, female adolescent)*.

“*…because they have more sexual urge” (R1, ADIKF, female adolescent)*.

“*I will say it is a girl because when the boy comes and says you this girl, I will want to have sex with you and if the girl is eventually in need of that she will agree.” (R4. ADIKF, female adolescent)*.

#### Respect for female sex partners

The majority of the respondents think that a boy does not respect a girl he has had sex with. The reasons are that; some think that virginity is the pride of girls; by having premarital sex the girl is seen to have lost her dignity; and the boy may dump the girl after having sex with her. See supporting quotes from male and female responses;

“*…because a girl's dignity is her virginity since the girl has sold her dignity and virginity to the guy it will make the boy treat the girl anyhow” (R11, ADAFM, male adolescent)*.

“*Yes, because they (males) have got what they want, then they will push you (girls) away” (R5, ADEZF, female adolescent)*.

“*Male counterparts lose respect for the females they have slept with because they feel you open up to everybody like that” (R 11, ADOHM, male adolescent)*.

#### Sexual urges

Many girls perceive that boys have more sexual urges and are not able to control themselves when compared to girls. Also, some mentioned that boys need more sex than girls. The following quotes buttress the responses;

“*It is the male that has more urge because when the girls are not dressing decently it will attract the male to have sex with them” (R3, ADABF, female adolescent)*.

“*…it is the boys that normally have the urge for having sex (R1, ADIKF female adolescent)*.

“*…the boys are under more pressure to have sex than girls because they always have sexual urge more than the girls” (R3, ADIKF, female adolescent)*.

#### Coerced or forced sex

The consensus is that girls should not be coerced into having sex. The reasons are because it is not good; the girl may not be in the mood for sex; coerced sex may cause physical injury; is likely to be unprotected with a high risk of infection; could result in an unwanted pregnancy. These responses are supported by the following quotes;

“*I said no. If the boy forces the girl and she gets pregnant, it is left for the boy to go and know how he will remove it, otherwise it will cause another problem* “(R5, ADIKM male adolescent).

“*You do not need to force the girl because if you force the girl, it may harm her”* (R2, ADIKM, male adolescent).

“*No, although sometimes the girls used to attract the boys by being naked (seductive dressing) on the road that should not concern the boys. They should not have to force the girls for sex*.” (R3, ADEZF, female adolescent).

However, a female respondent rightly points out that one of the reasons boys engage in forced sex is because they tend to use their masculine strength to overpower the girls.

“*Yes, the boys are used to forcing the girls but the girls don't want to have sex with them. If a girl and a boy are on the way and he told her let's have sex and she refuses he will force her because he has more power than her. (R4, ADEZF, female adolescent)*.

#### Peer pressure to have sex

The majority of the respondents agreed that peer pressure to have sex exists. Some indicated that both boys and girls are under pressure to have sex, but boys experience more peer pressure to have sex than girls. “…*the boys are under more pressure than the girls because they have more sexual urge than females*” (R3, ADIKF, female adolescent). Moreover, in the case of boys, their friends who have had sex before pressurize them to have sex by teasing, cajoling and calling them “…'*juu' guy, ‘middle man'*…” (R7, ADAFM, Male adolescent).

The pressure according to some male respondents is aggravated because boys are lured by the indecent dressing of girls when they are with them. “*Because if the girl wears indecent dressing like that, the boy will develop the sexual desire, then he will think of having sex with the girl*” (R1, ADIKM, male adolescent).

Concerning girls, some girls and boys reported that information from their peers, the material possessions their friends dangle before them and their family background are some of the factors that influence adolescent girls to have sex. Illustrative quotes follow;

“*They said that if you are going to have sex with the boy, he will be providing everything for you” (R8, ADEZF, female adolescent)*.

“*They said that if you have sex with a boy now when you get married it will not be hard for you” (R5, ADEZF, female adolescent)*.

“*It depends on the person's family upbringing. Material things shown by friends sometimes make girls join them” (R1, ADABF, female adolescent)*.

“*That females succumb to peer pressure to have sex “…because of [financial imbalance] lack of money” (R3, ADIKM, male adolescent)*.

#### Seductive dressing

The respondents said that indecent dressing such as exposing the body, wearing short skirts and showing their breasts, is seductive and contributes to the occurrence of coercive sex; and suggested that girls should dress decently to avoid sexual harassment. These suggestions were mostly opined by adolescent boys. See below for supporting quotes. “…*the reproductive system of the boys reacts more highly than the girls' when they see such dressing”* (R9, ADIKM, male adolescent).

“*Girls can lure men and if a man does not have the feeling to have sex, the girl's dressing can cause him to have the urge for sex” (R9, ADAFM, male adolescent)*.

“*The girl's dressing is not good. Like some girls like to wear miniskirts, or like the bra they use there is one they call push up, if they wear that one it would push their breast out and if the boys see it, they will feel like having sex with that person” (R5, ADIKM, male adolescent)*.

## Discussion

Our findings contribute to the knowledge of gender norms and ideologies about sexuality among adolescents, in the study site where about 9.6% of 15 to 19 years old girls have begun childbearing (NDHS, 2019). Our study demonstrates that norms and ideologies about sexuality prevail, are gendered, and are also influenced by socio-economic and demographic characteristics. This study is unique in that it examines variations among adolescents and generates findings that could be useful in designing strategies for addressing their sexual and reproductive health needs.

The prevailing beliefs of adolescents regarding sexuality are that abstinence should be upheld among unmarried adolescents, consent should be obtained before sexual intercourse, it is wrong for unmarried adolescents to have sex, and sex is permitted if contraceptives are used, in that order. These beliefs about sex among adolescents were sustained irrespective of place of residence, schooling, gender and wealth index. There were significant correlations between some of these ideologies and respondents' gender and place of residence. In qualitative findings, cultural norms, religious values and adverse health consequences of engaging in unprotected sexual intercourse were the reasons adolescents revealed for supporting premarital virginity. The implication is that gender ideology plays a large role in adolescents' decisions making concerning sexuality (Okigbo et al., 2018). Researchers in Ghana have also reported, that there is a dominant ideology of abstaining from premarital sex among young people (Van de Bongardt et al., 2015). However, this belief contradicts the observed sexual permissiveness among young people, which is influenced by modernization. Studies carried out in Nigeria, Tanzania and Vietnam have also reported that young people believe it is acceptable for adolescents to engage in premarital sex as long as they are not in school or are economically viable and that premarital sex is inevitable (Kagashe and Honest, 2013; Udigwe et al., 2014; Adogu et al., 2015; Bergenfeld et al., 2021). This affirms the result of these findings as adolescent boys than girls were more likely to believe that sex is permitted among adolescents as long as contraceptives are used.

Regarding gender norms about adolescent sexuality, the dominant ideology was that sex should be initiated by boys because girls feel shy initiating sex and that the boys experience more urge for sex than the girls. They also point out that the responsibility to ensure that sexual intercourse does not result in pregnancy lies on the girl/female partner. However, some adolescents feel that it is the responsibility of both the boys and girls to ensure that condoms are available before sexual intercourse to avoid unwanted pregnancy and STIs. This confirms findings from similar African research studies of gender norms about responsibility for preventing unwanted pregnancy (Capurchande et al., 2016; Nalukwago et al., 2019).

Although the majority of the adolescents in this survey believed that consent should be obtained before sex, a considerable number believed that a boy has to force a girl to have sex. However, the consensus in qualitative findings is that girls should not be coerced into having sex to avoid physical injury, infection and unwanted pregnancy. Some South African studies have reported that forced sex is considered as a sign of love, and is defensible if it occurs in the boy's house (Jewkes and Morrell, 2010; De Vries et al., 2014). These gender norms place the young boys (and indeed men) in the position of sexual dominance which in turn limits the capability of young girls to take control of their sexual and reproductive health (Pulerwitz et al., 2010). In our finding, a female categorically pointed out that one of the reasons boys engage in forced sex is because they tend to use their masculine strength to overpower the girls. Evidence shows that African men continue to uphold gender norms that confine women to conventional roles, including the role of sexual servants/slaves (El Feki et al., 2017). As young people observe and adopt these norms in their sexual relationships, it hinders the attainment of mutual and happy sexual relationships in adulthood. The prevalent gender norms about sexuality and contraception further highlight the need for culturally relevant community-led interventions/strategies designed on the background of an in-depth understanding of the social and cultural contexts, to contribute to changing gender narratives about sexuality.

Peer pressure to have sexual intercourse among adolescents exists. Although both boys and girls experience pressure to have sex, boys were described to experience more peer pressure to have sex than girls. Their friends who have had sexual intercourse, pressurize them to have sex by teasing and cajoling them. This corresponds with other findings which revealed that boys are more susceptible to peer influences as compared to girls (Bingenheimer et al., 2015; Widman et al., 2016). The pressure to have sex among boys could also be aggravated through the indecent/seductive dressing of girls when they are with boys. Hence, girls should dress decently to avoid sexual harassment and the occurrence of coercive sex. On the other hand, the information girls receive from their peers, the quest for material possessions and family socio-economic conditions contribute to girls' pressure to have sex.

Further analysis revealed that these norms and ideologies about sexual permissiveness are gendered. Adolescent boys were less likely to believe in premarital abstinence, and more likely to believe that premarital sexual intercourse is acceptable as long as contraception is assured. The belief that sex should be initiated by the boy alone was also gendered. The sexual permissiveness of adolescent boys and the intolerance of girls corroborate similar assertions that perceptions about sexuality, regardless of age group, are shaped by gender (Casique, 2019). Whilst, young boys express relatively more tolerance for pre-marital sexual relationships, adolescent girls primarily choose pre-marital sexual abstinence for the fear of the adverse social and health consequences of sexual intercourse (Long-Middleton et al., 2013). Our findings on gender ideologies and norms could be attributed to social and cultural environments which co-mingle to influence individual sexual behavioral choices, perceptions and actions (Hanson et al., 2014). Generally, adolescents learn what the society and culture expect of them from what their parents teach them, religious and cultural teachings, as well as other institutions of socialization (Okigbo et al., 2018; http://othersociologist.com/sociology-of-gender/). The findings highlight the importance of context in shaping the sexual ideologies of adolescents.

The scope of the study is limited because it only reports findings from unmarried adolescents aged 13 to 18 years. The exclusion of married adolescents and other age groups of adolescents limits the generalizability of the results. There is a need for further research of some of the progressive attitudes and beliefs which were found to be held more among adolescents. This will enable better understanding and approaches to address adolescents' needs based on gender and contextual differences. Another limitation of the study is that the use of FGDs may have limited participants' openness and contributions to discussions about sexuality. However, the researchers were trained to maintain a neutral attitude with participants and assured them of confidentiality of information they provided. The FGDs were used to promote dialogue and exchange on the beliefs that are commonly held by adolescents on gender and sexuality.

In conclusion, adolescents' beliefs about sexuality, including taking responsibility for the prevention of unwanted pregnancy, are influenced by norms and ideologies, some of which are gendered and correlate with the demographic and socio-economic characteristics of adolescents. Some of these ideologies/norms could be beneficial or harmless, while others could be harmful to adolescents' SRH. These harmful ideologies/norms about sexuality could contribute immensely in limiting adolescents from meeting their SRH needs with adverse implications on their health outcomes. Considering the potentially harmful effects of some of these norms and ideologies about sexuality, adolescent health programs/interventions should be tailored to address the identified gender norms and ideologies. Therefore, concerted efforts should target identified predictors of adolescents' beliefs about sexuality and other factors that could contribute to contextual changes.

Future research on ideologies/norms about adolescents' sexuality could be undertaken to understand the gendered perspectives of these adolescents and how their gendered perspectives interact with other socio-economic and socio-demographic factors such as age, schooling status, place of residence, peer or cultural influence and, working status. In order to determine similarities and peculiarities, studies could also be undertaken in other settings.

## Data availability statement

For the quantitative findings, the datasets presented in this study can be found in online repositories. The name of the repository and accession number(s) can be found below: UK Data Service https://doi.org/10.5255/UKDA-SN-854374.

## Ethics statement

The studies involving human participants were reviewed and approved by Health Research Ethics Committee of University of Nigeria Teaching Hospital with reference number NHREC/05/01/2008B-FWA00002458-IRB00002323. Written informed consent to participate in this study was provided by the participants' legal guardian/next of kin.

## Author contributions

CM, NE, and OO conceptualized and designed the study protocol and instruments used for data collection. IA, CM, UE, and IE were involved in data collection. IA and CM produced the first draft of the manuscript. All the authors participated in data analysis, reviewed, and approved the final version of the manuscript for journal submission.

## Funding

The results included in this manuscript are part of a research project that received funding from IDRC MENA+WA implementation research project on maternal and child health (IDRC Grant Number: 108677). However, the funder did not participate in designing the study, collecting and analyzing data, or writing and reviewing the manuscript.

## Conflict of interest

The authors declare that the research was conducted in the absence of any commercial or financial relationships that could be construed as a potential conflict of interest.

## Publisher's note

All claims expressed in this article are solely those of the authors and do not necessarily represent those of their affiliated organizations, or those of the publisher, the editors and the reviewers. Any product that may be evaluated in this article, or claim that may be made by its manufacturer, is not guaranteed or endorsed by the publisher.

## Author disclaimer

The views presented in this manuscript do not represent the funders' views and belong solely to the authors.
